# Perceptions of Academic Malpractice are not Always Biased by Halo Effects

**DOI:** 10.1177/00332941251317641

**Published:** 2025-01-27

**Authors:** Alexis Makin, Autumn Taylor, Lauren Macpherson

**Affiliations:** Department of Psychology, 4591 University of Liverpool, Liverpool, UK

**Keywords:** Halo effects, null results, academic malpractice, questionable research practices, facial attractiveness

## Abstract

The attractiveness halo effect has been discussed for over a century. Physically attractive people are often judged more favourably and accrue many life advantages. Halo effects have been observed in university settings for decades, but perhaps their influence is waning due to increased awareness of unconscious bias. The first study examined judgments of students accused of academic malpractice. Undergraduate student participants (*N* = 302) completed an online survey. They were presented with a vignette outlining a fictional but realistic academic malpractice scenario, beside a photograph of an attractive or unattractive ‘student’. Participants rated the fictional student in terms of guilt, appropriate punishment, and seriousness of malpractice. There was no evidence for halo effects. The second study examined judgements of fictional researchers accused of questionable research practices. Psychology researchers (*N* = 42) completed another online survey. They were presented with a vignette describing dubious data manipulation, beside a photo of an attractive or unattractive ‘researcher’. The same rating scales were used, and again, there were no halo effects. Evidently, university students and staff can sometimes make professional judgements without emotional bias. These null results are important, because they show that halo effects may not now be so pervasive.

## Introduction

The *Halo effect* was first discussed by Thorndike (1920) during his time in the US military. Thorndike asked commanding officers to rate their subordinates on physical appearance, leadership, loyalty, and intelligence. Despite not knowing their subordinates personally, officers’ perceptions were based almost solely on physical appearance, and subordinates who were rated as more attractive were also rated higher on other desirable traits.

The halo effect is now a standard entry in psychology textbooks, and it is apparently very robust and widespread. A meta-analysis conducted by Langlois et al. (2000) found a cross-cultural agreement on physical attractiveness, and evidence for halo effect worldwide. Batres and Shiramizu (2022) confirmed this. Across 11 global regions, male and female faces which were deemed more attractive were also seen as more confident, intelligent, sociable, responsible, trustworthy, and emotionally stable.

This ‘lookism’ is a potential problem in real world settings, such as criminal justice, recruitment, and education. Physically attractive people may accrue ‘unfair’ life advantages in all these domains, as well as having more sexual and romantic opportunity. The vast halo effect literature provides numerous examples. For instance, unattractive people receive 36% fewer call backs after an interview (Boo et al., 2012) and are more likely to have their employment terminated (Commisso & Finkelstein, 2012). Watkins and Johnston (2002) found that when CVs were of a mediocre quality, those paired with a photo of an attractive candidate were rated more favourably than those without photos. Finally, attractive individuals often receive less severe sentencing from jurors (Desantts & Kayson, 1997; Stewart, 1980).

Halo effects are still observed in the most recent studies. Schreiber et al. (2024) asked experienced male investors to evaluate an identical business pitch delivered by either an averagely attractive female actress or a highly attractive female actress. The highly attractive actress raised the participant’s cortisol levels, and she was rated as more competent, and her business plan was evaluated more positively. In another recent study the results were more complicated. Han and Laurent (2023) found that the standard beautiful = morally good association can be offset by (1) a beautiful = vain association, and then (2) a vain = morally bad association.

There is some evidence that halo effects bias both student evaluation of university teachers (Felton et al., 2008) and teacher evaluation of students (Cipriani & Zago, 2011). This can extend to judgements of academic malpractice. Efran (1974) simulated student-faculty courtroom to address cases of faculty misconduct. In a mock trial regarding exam cheating, the defendant who was deemed more physically attractive was perceived as less culpable and received a less severe punishment from student jurors. The overarching message of such studies is that halo effects can lead to social injustices, and people should effortfully counteract their own biases when performing responsible roles.

This project revisited the question of whether halo effects bias judgments of academic malpractice. Student malpractice was examined in study 1, and staff research malpractice in study 2. Such judgements are made frequently in modern university settings, so it is important to assess whether they are subject to problematic biases.

## Study 1 Introduction

### Halo Effects and Student Academic Malpractice

Study 1 examined halo effect influences on judgements of student academic malpractice. One possibility is that public awareness of bias and prejudice has increased, so people are now immune to halo effects. However, it is likely that halo effects are still prevalent. It was thus hypothesized that more attractive students would be judged more favourably in terms of guilt, appropriate punishment, and seriousness of malpractice.

Specifically, there were four hypotheses. (1) Participants in the attractive student condition would rate the student as less guilty. (2) Participants in the attractive student condition would recommend a more lenient punishment. (3) Participants in the attractive student condition would rate the academic malpractice accusation as less serious. (4) Participants in the attractive student condition would rate the student as more attractive.

### Study 1 Method

Study 1 used a between-subjects online survey on the Qualtrics platform. A sample 302 students were presented with a vignette paired with a picture of a face (Figure 1). The participants were randomly allocated to an attractive or unattractive condition. Faces were obtained from the open-source Chicago Face Database (https://www.chicagofaces.org/). This resource also provides mean attractiveness ratings from over 1000 participants from diverse backgrounds (Ma et al., 2015). Therefore, faces that are considered attractive or unattractive by most people could be selected. Attractiveness differences were also confirmed by the participants, validating the chosen stimuli.

**Figure 1.**
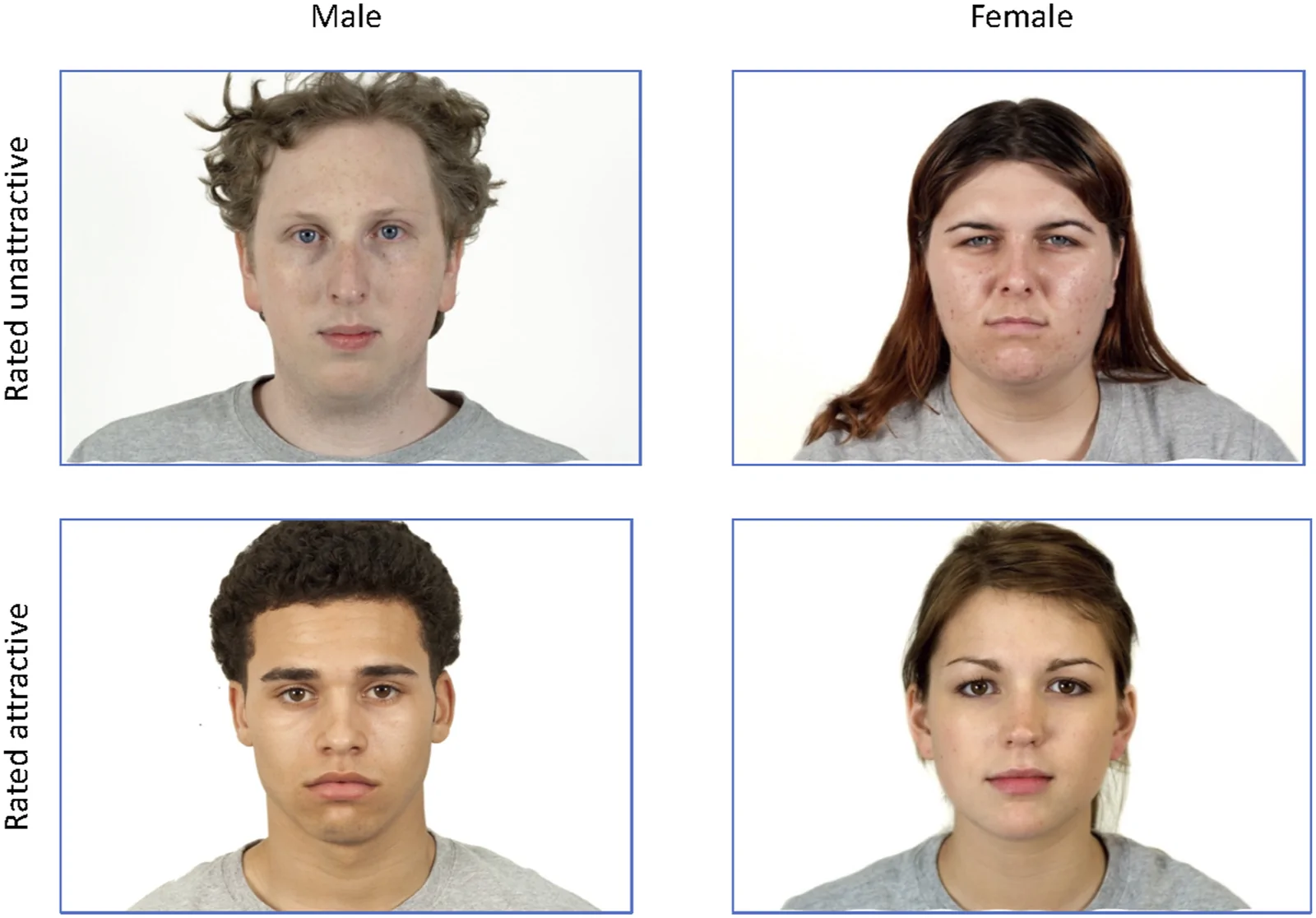
Fictional student faces used in Study 1. These were obtained from the Chicago face database (https://www.chicagofaces.org/). These were codenamed CFD-WF-022-017-N, CFD-WF-210-086-N, CFD-WM-206-045-N and CFD-WM-250-157-N.

The vignette besides the face described realistic case of relatively minor academic malpractice:

“This is Harry [Emily] Smith, an undergraduate student studying Psychology at the University of Liverpool. Harry [Emily] has previously submitted his work late but has never been accused of academic malpractice. His [Her] current average grade is 65.2%. In year 1, Harry [Emily] reported struggling with procrastination and some mental health issues, but never applied for mitigating circumstances. In a recent essay on the neural basis of memory, Turnitin identified 50% similarity with a published review article (although there were no long stretches of identical text). There were also some problems with attributing quotes, so quotes from another paper appeared without proper acknowledgement. Finally, the essay was 10% over the word count, even though the title page suggested it was within the word limit.”

Half the participants were presented with an attractive face (*N* = 151), half with an unattractive face (*N* = 151). Male participants received female faces, and female participants received male faces. Only participants self-identifying as heterosexual or bisexual were included in analysis, so all could potentially be attracted to the presented face. In the female face condition, the name Harry was replaced with Emily. Trial structure is shown in Figure 2. Participants rated ‘Harry’ or ‘Emily’ on four dimensions: guilt, punishment, seriousness, and attractiveness.

**Figure 2.**
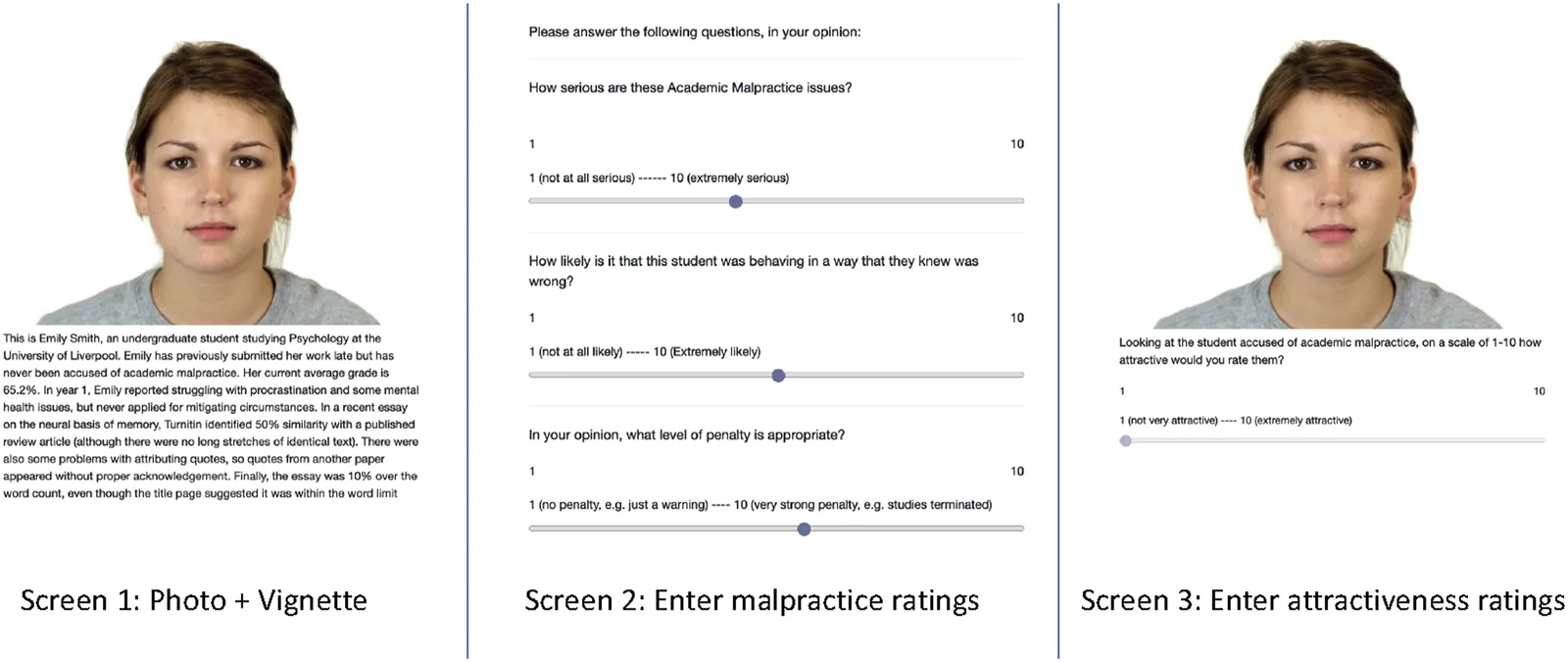
Trial structure in study 1.

To assess guilt, the question was:

How likely is it that the student was behaving in a way that they knew was wrong?’ (1 = not at all likely, 10 = extremely likely).

To assess punishment:

‘In your opinion, what level of penalty is appropriate?’ (1 = no penalty, e.g. just a warning, 10 = very strong penalty, e.g. termination of studies).

To assess seriousness:

How serious are these academic malpractice issues? (1 = not serious at all, 10 = extremely serious).

To assess attractiveness:

Looking at the student accused of academic malpractice, how attractive would you rate them?’ (1 = not very attractive, 10 = extremely attractive).

Participants were recruited from the undergraduate psychology student population at the University of Liverpool, UK. Fifteen participants were excluded because they ticked the ‘other’, ‘prefer not to say’ or ‘homosexual’ option in response to their sexual orientation. This left 302 valid datasets. There were 274 female participants and 28 male participants (mean age 18.8, range 18–48).

### Study 1 Results

Mean attractiveness ratings were comparable to the norming data supplied by Chicago face database (when standardized as a percentage of maximum possible score): Attractive male (59 vs. 59%); Unattractive male (26 vs. 23%); Attractive female (73 vs. 58%); Unattractive female (27 vs. 22%).

Contrary to the study’s hypotheses, there were no effects of facial attractiveness on any of the academic malpractice DVs, despite large differences in subjective attractiveness (Figure 3(A)). This was first analysed with four separate independent samples t tests on guilt (t (300) = 0.154, *p* = .878, Cohen’s d_s_ = 0.018), punishment (t (300) = 0.244, *p* = .808, d_s_ = 0.028), seriousness (t (300) = 0.461, *p* = .645, d_s_ = 0.053) and attractiveness (t (265.22) = 18.586, *p* < .001, d_s_ = 2.139, DF corrected for violation of homogeneity of variance).

**Figure 3.**
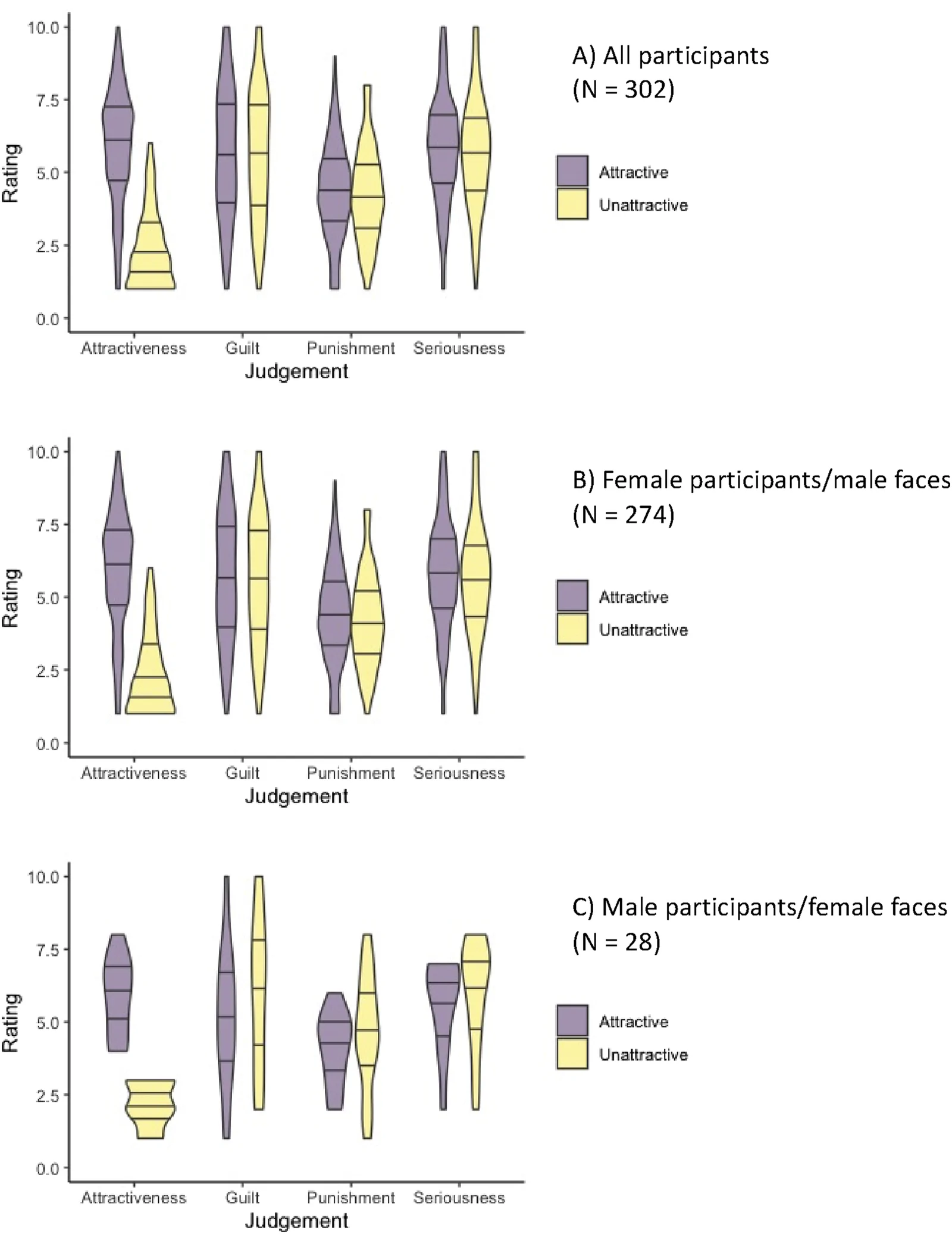
Results of Study 1. Violin plots show the distribution of datapoints and 25, 50 and 75% lines. Participants agreed with the norming data from the Chicago face database: They rated the ‘attractive’ faces as more attractive (left side). However, facial attraction had no effect on guilt, punishment, and seriousness ratings. Results were similar for all participants (a) female participants (b) and male participants (c).

Non-significant *p* values from these t tests should be interpreted as absence of evidence, not evidence of absence: They do not confirm the null hypothesis. Bayesian t tests were thus used to determine whether the data supports the null hypothesis (H0, no halo effects) or the alternative hypothesis (H1, halo effects). Bayes factors provide modest evidence in favour of H0 for guilt (BF01 = 7.806), and punishment (BF01 = 7.674) and seriousness (BF01 = 7.132) (BF01 = 3 is the conventional threshold for modest evidence when updating an uninformed prior).

There were more female participants (274) than male participants (28) in Study 1. This limitation notwithstanding, it should be noted that there were no interactions between Attractiveness and Participant gender (largest interaction effect F (1,298) = 1.139, *p* = .287, partial η2 = 0.004). Furthermore, results were very similar when data was split by participant gender (Figure 3(b) and (c)). There were no significant halo effects on any DV in either group (largest effect; t (26) = 0.921, *p* = .366, d_s_ = 0.349). Bayesian t tests could not provide strong support for H0 in the male subgroup, BF01 ranged from 2.059 to 2.598. In the larger female subgroup, BF01 ranged from 6.265 to 6.836.

## Study 1 Discussion

There were no halo effects in Study 1. The fictional attractive students were not rated as less guilty, or less deserving of punishment, or as having committed a less serious offence. Results were similar for male and female participants, although unequal sample sizes compromise statistical comparison. The null results cannot be explained by floor or ceiling effects, as average ratings were around the centre of the scales.

## Study 2 Introduction

Students are not the only people at university known to engage in malpractice: Their research-active teachers are not perfect. The last decade has seen increasing awareness of *questionable research practices* (QRPs) (Bishop, 2019; John et al., 2012; Munafò et al., 2017). Academics know the career value of publication, and they know that statistical significance gates acceptance rate. Obtaining *p* < .05 is good news for careers. There are many flexible points in a typical analysis pipeline that can be exploited to nudge borderline effects across the chosen significance threshold (*p*-value hacking). It is then possible to craft a narrative around the significant effects, giving the impression they were hypothesised a priori. This is called ‘Hypothesising After Results Known’ (‘HARKing’ for short). Most worrying, a combination of p-hacking and HARKing can turn noise into a theory, which may then persist in the literature for decades, especially if subsequent failures to replicate remain in the file drawer (Kerr, 1998). Most researchers disapprove of QRPs because they undermine scientific integrity. But are judgments about the severity of fictional QRPs biased by halo effects?

### Study 2 Method

Study 2 was like Study 1, but a sample of academic psychologists from the University of Liverpool was recruited. The attractive and unattractive fictional ‘researchers’ were also taken from the Chicago face database. The faces are not reproduced here but can be view on https://www.chicagofaces.org/. Codes were CFD-WF-206-147-N, CFD-WF-026-002-N, CFD-WM-029-023-N, CFD-WM-215-041-N.

The vignette described several QRPs, including post hoc attempts to make an effect significant, selective termination, data-dipping and HARKing:

An early career academic named (Robert [Emily] Smith, pictured) is at the University of Liverpool Psychology department. They have had a successful career studying cognitive psychology and published 10 papers. In a recent study, the effect of mood on memory performance was borderline significant (p = .06). This non-significant effect was disappointing because the experiment was thus inconclusive. They decided to re-examine their data while removing outliers >2.5 SD from the mean, and with gender included as a between-subject’s factor. The results were still borderline significant (p = .055). The researchers then realized that the ages of the participants were not normally distributed, so they made a post hoc decision to removed participants over 40 and replace them. After each new participant was added, they checked to see if this had made the effect of mood significant. Finally, after further exploratory analysis (with the original, older participants re-included) they found a significant effect of mood on memory, but only in the female participants (p = .026, one tailed, N = 30) but not the male participants (p = .091, one tailed, N = 20). The researcher wrote a simplified manuscript giving the impression that the observed effects were predicted a priori.

As a minor improvement on Study 1, the number of increments on the response scale was increased to 100. This allowed participants to report more refined judgements. Study 2 only had 42 participants (7 male, aged 22 to 61, M = 38.62). There were 21 participants in the attractive condition and 21 in the unattractive condition. Study 2 used the same guilt, punishment, seriousness, and attractiveness ratings as Study 1 (but changed the phrase ‘studies terminated’ to ‘employment terminated’). Again, homosexual participants were excluded, and males received female faces and vice versa. Therefore, all participants were presented with faces they could potentially find attractive.

### Study 2 Results

As with study 1, mean attractiveness ratings were comparable to the norming data supplied by Chicago face database (when standardized as a percentage of maximum possible score): Attractive male (66 vs. 55%); Unattractive male (27 vs. 27%); Attractive female (65 vs. 61%); Unattractive female (25 vs. 21%).

Results of study 2 are shown in Figure 4. Participants evidently disapproved of the QRPs described in the vignette, with average ratings over 50. They also had rated the face in the attractive condition as more attractive (t (40) = 6.199, *p* < .001, d_s_ = 1.913, BF10 > 1000). As with Study 1, there was no effect of Attractiveness on judgements of guilt (t (40) = −0.481, *p* = .633, d_s_ = −0.149, BF01 = 3.007), punishment (t (40) = −0.053, *p* = .958, d_s_ = −0.016, BF01 = 3.297) or seriousness (t (40) = 0.572, *p* = .571, d_s_ = 0.176, BF01 = 2.895). There were not enough male participants to subdivide results by participant gender.

**Figure 4.**
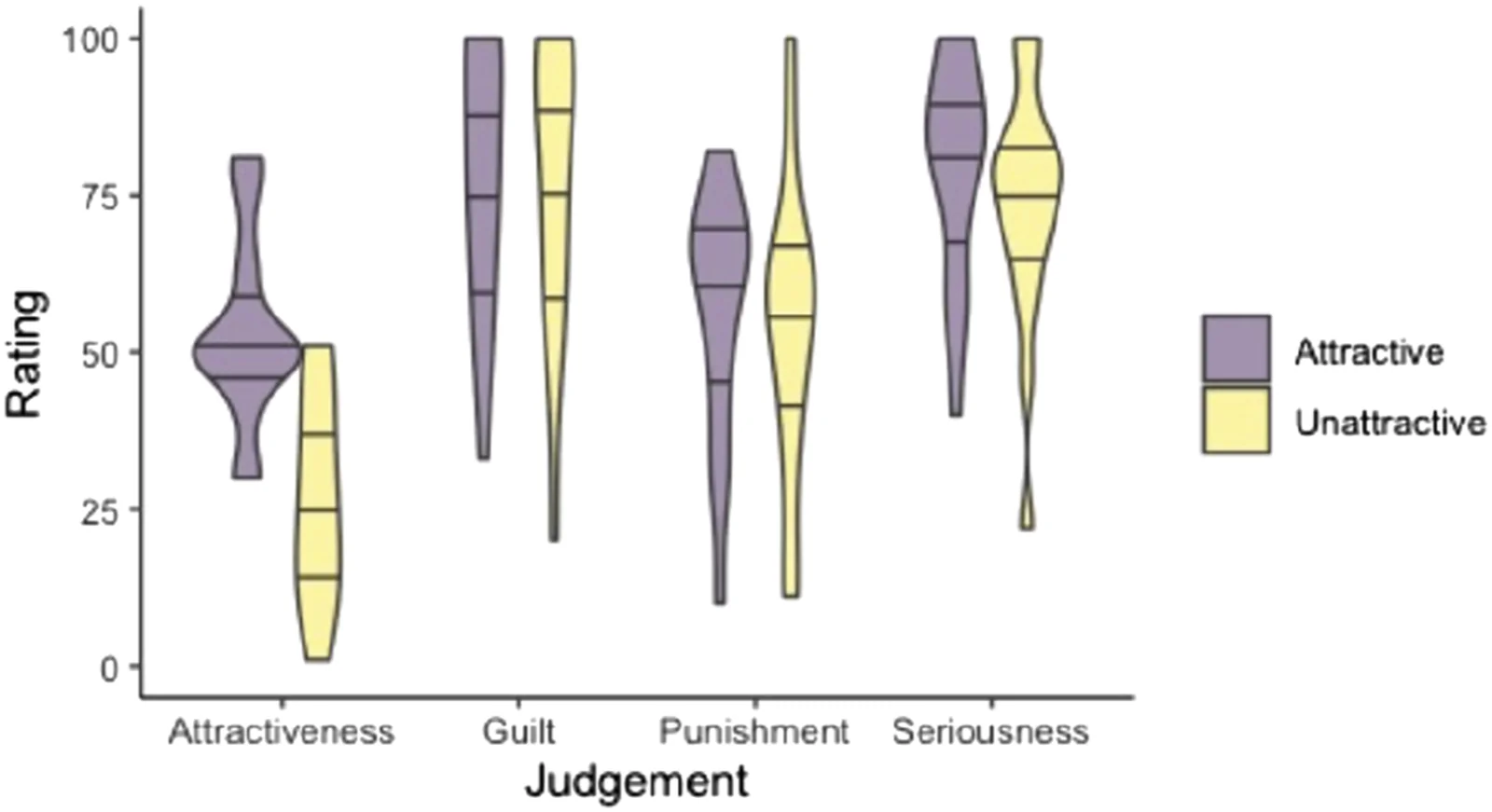
Results of study 2. Conventions are the same as Figure 3(a).

## Study 2 Discussion

Study 2 replicated the null results of study 1 in a different context. There was no effect of attractiveness on judgements about questionable research practice. This was despite substantial difference between the faces on attractiveness ratings, and no evidence of floor or ceiling effects.

## General Discussion

The two studies found no evidence for halo effects on realistic judgments of academic malpractice by a majority female participation pool. Students were no more lenient towards attractive students, and researchers were no more lenient towards attractive researchers. While the participants found the ‘attractive’ faces from Chicago face database to be more attractive, this attraction did not bias judgments about plagiarism (Study 1) or questionable research practices (Study 2).

Absent halo effects are newsworthy for two reasons. This null result cannot be dismissed as a floor or ceiling effect – judgements of guilt, punishment and seriousness were not compressed at extreme ends of the scale. Furthermore, the null result cannot be dismissed because of low statistical power. A relatively small between subjects’ effect could easily be missed in a study with a relatively small sample. However, the sample was large in Study 1. Moreover, Bayesian independent samples t tests provided evidence of absence, and not merely absence of evidence, in both studies (except for seriousness ratings in Study 2, which did not reach the conventional Bayes factor threshold).

This work highlights the importance of publishing null results. It is possible that every time a halo effect is present, the data is published, but every time halo effect is absent, the data remains in the file drawer. A meta-analysis on halo effects would use an unrepresentative subset of studies that ‘worked’. Any reader would come away with the erroneous impression that halo effects are hyper-insidious and near-exceptionless. This is a realistic concern: publication bias is widespread in social science (Franco et al., 2014).

Admittedly, this research would have been more interesting if halo effects had been found, but that interest-asymmetry is part of the problem. If ‘boring’ null results remain invisible, the scientific record will systematically overestimate the strength of effects, and this cannot be corrected by failed replications. In one influential commentary, Laws (2013) is pessimistic about claims that psychology is self-correcting. He argues that researchers need to show leadership, but:

“This leadership will however require psychologists to take a more active role in submitting replications and null findings—science is clearly not self-correcting.”

(Page 7)

He further recommends that ‘failed’ experiments should not be marginalized in specialist null results journals:

“Although laudable, such journals create a special space for replications and null findings rather than acknowledging their place in the centre of science.”

(Page 7)

## Limitations and Future Research

This research had several limitations. It could be that halo effects are stronger in real life, where there is an opportunity to interact with people and impress them. A real person is a far richer stimulus than a static photograph. However, this method used here is typical of halo effect literature (Landy & Sigall, 1974; Lenoir & Stocks, 2019; Watkins & Johnston, 2002). Whether the current results would be obtained in research with alternative methodology is an empirical question.

One limitation is that the vignettes and faces were not present on the screen while participants entered their judgements (Figure 2). It could be that facial attraction did, in fact, bias impressions while reading the vignette, but then the bias faded rapidly before entering judgments. However, if there was a substantial halo effect, it would probably not decay so quickly.

It is also possible that participants paid no attention to the face. However, this is also unlikely. Faces are very salient compared to text, and they are evaluated immediately and automatically. Moreover, the vignette encouraged participants to interpret the face as an illustration of the character described in the story (e.g., ‘This is Harry Smith…’).

Although variations on the design may give different results, the task did resemble a real malpractice evaluation scenario. A typical student database features photographs alongside contact details and grades. When considering cases of academic malpractice, the responsible staff member may check the student database (and see the photograph), before completing the malpractice form in different tab.

It might also be that male participants are more prone to halo effects (Efran, 1974), and the sample was predominantly female (in study 1, this was a consequence of sampling from the undergraduate psychology student population, who are mostly female). However, the small subset of males in Study 1 had very similar results to the larger subset of females. It remains possible that a future study with a large sample of males would detect halo effects.

Critics might also note that the three questions were not conceptually independent. For instance, somebody’s assessment of appropriate punishment might reflect both the seriousness of the crime and the intentions of the criminal. One cannot be sure whether the participants considered such interdependencies. However, the variables were positively correlated: Participants who gave high guilt scores also gave high punishment and seriousness scores (Figure 5). There may be a latent variable - ‘blameworthiness’ - which contributes to all three. However, halo effects did not bias subjective blameworthiness.

**Figure 5.**
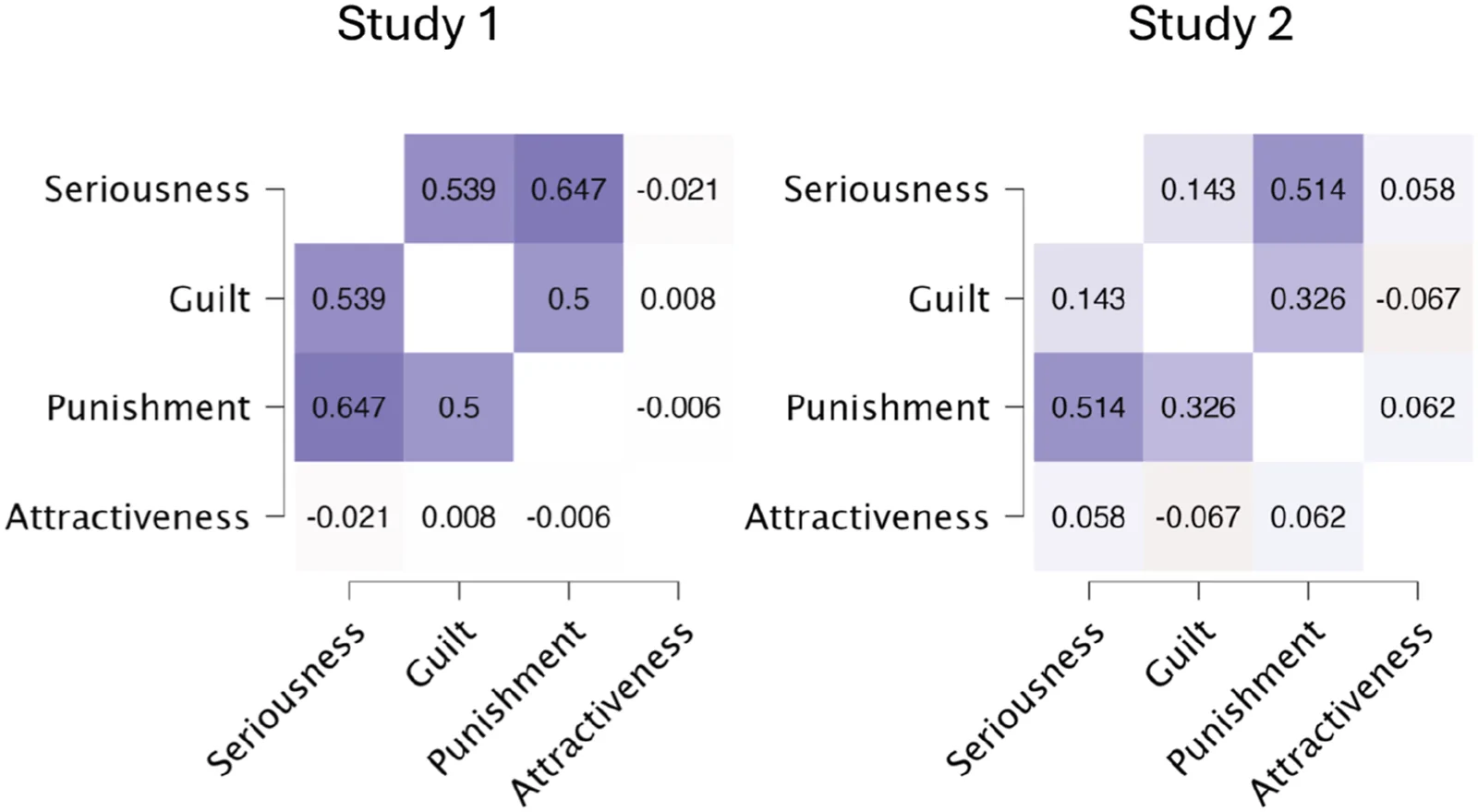
Correlations between judgments. Results from study 1 are shown on the left, and results from study 2 are shown on the right. Each cell gives a correlation coefficient, colour coded blue for positive and red for negative. Participants who entered high seriousness scores also entered higher guilt and punishment scores (all *p* < .001 in study 1, punishment vs. seriousness *p* < .001, punishment vs. guilt *p* = .035, seriousness vs. guilt *p* = .365 in study 2). Attractiveness judgements did not correlate with other variables (all *p* > .673).

While these null results may seem inconsistent with the wider halo effect literature, they are consistent with previous research on halo effects and moral goodness. A meta-analysis by Eagly et al. (1991) found that halo effects are largest for judgements social competence, intermediate for potency, adjustment and intellectual competence, and around zero for integrity and concern for others. More recently, Han and Laurent (2023) again found that attractive people are *not* consistently rated as morally superior. This is possibly because beauty is associated both vanity and virtue. Academic integrity is arguably a form of moral goodness, so the same balance of associations could mask halo effects in this domain too.

## Conclusion

We tentatively predicted that evaluations of academic integrity would be subject to attractiveness halo effects. There was no evidence for this. Attractive students engaging in academic malpractice were not judged more favourably in study 1, and attractive researchers engaging in questionable research practice were not judged more favourably in study 2. As well as combatting publication bias, the current study has a positive message and an applied dimension. It suggests halo effects are not inevitable, and that they can be overcome. One possibility is that people are now more aware of their own potential unconscious biases, and consciously seek to discount them in the name of objectivity and fairness. Future work should test whether halo effects can indeed be reduced by self-awareness. Another possibility is that halo effects were never ubiquitous in earlier eras. There may be many scenarios where people can, in fact, focus on a cognitive task without emotional bias. Undoubtedly emotional biases happen often, but perhaps not as often as we fear.

## Data Availability

All data associated with this study, along with R codes for data analysis, are freely available on open science framework (https://osf.io/tfz4r/).
